# ‘Sick and tired’: Patients reported reasons for not participating in clinical psychiatric research

**DOI:** 10.1111/hex.12977

**Published:** 2019-10-11

**Authors:** Liv Bixo, Janet L. Cunningham, Lisa Ekselius, Caisa Öster, Mia Ramklint

**Affiliations:** ^1^ Department of Neuroscience, Psychiatry Uppsala University Uppsala Sweden

**Keywords:** psychiatry, research participation

## Abstract

**Background:**

Meaningful and generalizable research depends on patients' willingness to participate. Studies often fail to reach satisfactory representativeness.

**Objective:**

This paper aims to investigate reasons for not participating in research among young adult patients with psychiatric illness.

**Method:**

A quantitative cross‐sectional study was performed based on questionnaires reported on by 51 psychiatric patients (14 males, 35 females and two unspecified) who had previously declined participation in an ongoing research project. Thereafter, a qualitative interview with subsequent content analysis was conducted with ten additional patients (five males, five females).

**Results:**

The questionnaires indicate being ‘too tired/too sick to participate’ as the most common barrier. Lack of time and fear of needles were other common barriers. Lack of trust or belief in the value of research was less inhibitive. In the interviews, disabling psychiatric symptoms were confirmed as the main reason for not participating. Several potential ways to increase participation were identified, such as simplification of procedures and information as well as providing rewards and feedback, and building relationships before asking.

**Conclusion:**

This study is unusual as it focuses on the group of young people attending psychiatry outpatient clinics we know very little about – those who do not partake in research. Our results indicate that fatigue and sickness reduce research participation and identify factors that may facilitate enrolment of this important group.

## 1 INTRODUCTION

Patients' willingness to participate in clinical research is crucial for producing meaningful and generalizable results.^1^, ^2^ Today, many studies fail to reach representativeness,^3^, ^4^ regardless of study design, country or field of research.^5^, ^6^ For example, a review found that only a third of the original target population were recruited in randomized, controlled multicenter studies performed in the UK between 1994 and 2002.^7^ In addition to the risk of misleading outcomes,^8^ scientists are also often forced to cancel research in advance due to an insufficient number of participants.^6^ Participant recruitment in psychiatric research is particularly unsatisfying.^9^, ^10^ For example, only five to ten percentage of patients screened for affective and schizophrenic disorders in large multicenter trials agreed to participate.^10^

In order to tackle research recruitment problems, knowledge of what influences study participation is needed. Regarding research in general, the decision to participate is often based on altruistic or personal reasons.^11^ Age has an impact: elderly people tend to decline to participate more frequently, possibly due to the recruitment process and study design not being optimized for senior participants with regard to potential vision, hearing and physical disabilities.^8^, ^12^ Ethnicity, sociodemographic and socioeconomic factors have been proposed to have an impact, but data are inconclusive.^13^, ^14^, ^15^, ^16^ Expectations of better treatment and concerns about health in general as well as extensive disease duration prior to the request, and experiences with ineffective treatments also increase research enrolment.^17^ Conversely, hesitations are often based on concerns about injury or practical inconvenience such as time expenditure,^18^ and these reservations seem to have a greater impact than motivating factors. In regard to biobanks, attitudes are predominantly positive. Participants often feel that their contribution is important and will benefit both themselves and others.^19^, ^20^, ^21^ Concerns about insufficient data protection^22^ and invasive sampling methods affect participation negatively.^23^ Information concerning study outcomes is appreciated.^24^

In clinical psychiatric studies, patients suffering from mental illness also report a positive attitude towards research.^25^, ^26^, ^27^ Expectations of obtaining more consistent follow‐up and being a ‘special patient’ rather than just a name in the system have been described as motivating factors.^28^ Similar to medical research in general, altruistic factors,^29^ insight and trust in research, convenient demands of participation, ‘biological benefits’ and receiving some kind of reward also lead to positive attitudes towards participation.^16^ Participants with mental illness also show great interest in being informed of the study results.^26^ The opposites of the above‐mentioned factors, lack of trust, the burden of participation and lack of compensation or benefits reduce the willingness to take part.^16^ The stigma entailed by psychiatric diseases may also affect research participation negatively.^2^ Patients with mental illness are considered particularly vulnerable to ask about research participation, since psychiatric diseases may influence decision‐making ability and the process of informed consent.^30^, ^31^ Some research methods might also contribute to increased stress (such as disclosure of personal information in a group context).^32^ However, studies specifically asking those who declined to participate are few.^33^, ^34^

This study aims to investigate the reasons why young people attending psychiatry outpatient clinics decline to participate in clinical research, including biobanking, and to examine whether and how their rejection could have been changed. This knowledge may be used to facilitate patient participation, especially for those with most symptoms, and thereby increase representativeness in psychiatric research.

### 1.1 Ethical permission

Since this study included psychiatric patients who had previously declined research, participation ethical issues were raised. Two dimensions of vulnerability, capacity and voluntariness, were considered.^35^ Patients were invited when they already had an ongoing treatment and were clearly informed that this would not be influenced by their decision. The study received ethical permission from the regional ethics board in Uppsala (Reg. no. 2012/081 and Reg. no. 2016/412).

## 2 METHOD

### 2.1 Study design

This report contains a quantitative cross‐sectional study based on questionnaires and a qualitative interview study with subsequent content analysis.

### 2.2 Study sample

Uppsala Psychiatric Patient Sample (UPP) is an infrastructure for data collection and biobanking within the psychiatric clinic at Uppsala University hospital.^25^ All new patients, aged 18‐25, at the psychiatric unit for young adults are consecutively asked during their first visit at the clinic to participate in UPP by contributing data and biological samples (ie blood, saliva, etc). In 2015, when more than 1100 patients had been approached, 41.4% had chosen to take part. In 2016, we wanted to approach the group who had denied participation, and UPP rejectors needed to be identified, but there was no register over non‐participants for the current study. Therefore, recruitment occurred during an ordinary visit to the clinic. The patients were approached by their regular contact, who was instructed to ask the patient whether he/she had participated in UPP. Patients who reported that they had chosen not to participate in UPP were then asked whether they would consider contributing knowledge about why people choose not to participate in research, by responding to a questionnaire. The number of patients approached was not registered. Participants completed the questionnaire anonymously, set it in an envelope and dropped it in a locked mailbox. That was regarded as informed consent to participate in the quantitative study with preserved anonymity. Patients were recruited during the period of February to November 2016. In total, 51 patients, 35 (68.5%) women, 14 (27.5%) men and two (3.9%), who did not specify sex, completed the questionnaire. The time passed between declining participation in UPP and being asked about participating in this study was not recorded, but could vary from months to years.

In addition, a qualitative interview was performed with ten additional patients, five women and five men. After using the same recruitment procedure and information about the interview study had been given orally by their regular contact, interested participants were contacted by phone and given the opportunity to ask questions. Written consent was then collected for the interview study by the interviewer. The sample size was not adjusted based on analysis of saturation. Instead, ten individuals, all patients during the period of recruitment that choose to take part in the interview, were included.

### 2.3 Data collection

#### 2.3.1 Questionnaires

The questionnaire was constructed as statements, based on the most commonly reported reasons to decline research participation.^18^, ^22^, ^23^, ^33^, ^34^, ^36^ After a literature review, two of the authors (MR and CÖ) discussed the findings and selected the questions. The statements are not part of a general construct, and there was no theory about their relationship to each other. The respondents were asked to grade his/her agreement with each statement on a Likert scale from ‘1’ to ‘5’, where ‘1’ represented no agreement at all and ‘5’ total agreement. The statements examined both practical reasons for rejecting research participation, such as lack of time and fear of needles, and general attitudes towards research (for all questions, see Table 1). The questionnaire was not previously used in any research project, and no pilot was performed. Within this study group, the internal consistency was low, and Cronbach's alpha was .335.

**Table 1:** Consensus ratio for reasons not to participate in research among patients with psychiatric illness (n = 51)

Consensus ratio	1	2	3	4	5
	I do not agree at all		I agree to some level		I agree completely
Questions	n (%)	n (%)	n (%)	n (%)	n (%)
Q1 I would have needed more information to participate in this project	25 (49.0)	5 (9.8)	12 (23.5)	3 (5.9)	6 (11.8)
Q2 It takes too much time to participate in this research project	10 (19.6)	6 (11.8)	18 (35.3)	8 (15.7)	9 (17.6)
Q3 I'm too sick/tired to get involved in this project	7 (13.7)	6 (11.8)	13 (25.5)	10 (19.6)	15 (29.4)
Q4 I do not think this project will provide valuable knowledge	31 (60.8)	10 (19.6)	8 (15.7)	0 (0)	2 (3.9)
Q5 I can imagine that I will be part of another research project in the future	7 (13.7)	7 (13.7)	19 (37.3)	5 (9.8)	13 (25.5)
Q6 I never participate in research	21 (41.2)	6 (11.8)	12 (23.5)	6 (11.8)	6 (11.8)
Q7 I do not trust that information about me stays within the research group	28 (54.9)	10 (19.6)	6 (11.8)	3 (5.9)	4 (7.8)
Q8a Because: I'm afraid of injection needles, I don't want to have blood tests performed	24 (47.1)	4 (7.8)	9 (17.6)	4 (7.8)	10 (19.6)
Q8b I'm afraid that the blood sample may get into the wrong hands	38 (74.5)	3 (5.9)	5 (9.8)	1 (2.0)	4 (7.8)

#### 2.3.2 Qualitative interviews

A non‐standardized, semi‐structured interview guide was constructed for this study. The questions were drafted from six predetermined categories since the aim was to explore whether previous findings in research are present in this sample: reasons to not participate in research^8^, ^37^; reasons to participate in research^14^, ^17^; changeable barriers to research participation^9^, ^36^; general attitudes and thoughts towards research^28^, ^29^; attitudes and thoughts towards psychiatric research^25^, ^26^; and attitudes and thoughts towards biobanks.^21^, ^24^ The interviews were performed at the clinic by the first author (LB) who did not participate in the care of the patients. The dialogue was recorded, and afterwards, audio files were anonymized and transcribed into a total of 92 pages of text.

### 2.4 Analysis of collected interview data

The transcripts were further analysed by content analysis, with a manifest and deductive approach.^38^ The first author (LB), a medical student with previous internship in psychiatric care, carried out the analysis, and classification was then scrutinized, discussed and completed together with one of the other authors (CÖ), a researcher with long experience of qualitative analysis and extensive practice in the field of psychiatric care. Both authors read the transcripts several times. Units of meaning, identified from sentences or paragraphs and rigorously considered representative to the context, were extracted, condensed and sorted into codes. The codes were classified into one of the predetermined categories mentioned above. The categories were then divided into subcategories based on dissimilarities within the categories. The analysis continued until all subcategories were considered clearly defined and distinct from one another.^38^

### 2.5 Statistical analysis

For reliability analyses, internal consistency was measured by Cronbach's alpha and an inter‐item correlation matrix was performed. Men and women were compared using the Mann‐Whitney test. Data were analysed by the statistical program SPSS, version 24.

## 3 RESULTS

### 3.1 Quantitative cross‐sectional study

The respondents' graded agreements in the questionnaires are presented in Table 1.

The most common reason to not take part in research was ‘I am too sick/tired to participate’.

Men and women did not show any significant difference in degree of agreement concerning any reasons included in the questionnaire. An inter‐item correlation matrix revealed low correlations (<0.4), except for Q7 and Q8 – ‘I do not trust information about me stays within the research group’ and ‘I'm afraid that the blood sample may get into the wrong hands’ (0.667) and between Q2 and Q3 – ‘It takes to much time to participate in this research’ and ‘ I'm too sick/tired to get involved in this project’ (0.441) and a negative correlation between Q5 and Q6 – ‘I can imagine that I will be part of another research project in the future’ and ‘I never participate in research’ (−0.428).

### 3.2 Qualitative study

Table 2 presents the six categories together with subcategories and codes that are derived from interviews. Each category and the subcategories (in italics) are described below and illustrated with quotes.

**Table 2:** Qualitative analysis of ten interviews about psychiatric clinical research performed with five men and five women who had declined research participation

Category	Subcategory	Codes
1. Reasons not to participate in research	Affected by disease	Loss of energy due to disease Social anxiety Planning difficulties (due to ADHD)
	Participation takes time	Haven't got the time Other priorities Too stressed Too many things to focus on
	Uncomfortable	Fear of leaving blood samples Lack of trust in research methods Concern of handing out personal data The invitation to research felt offensive Physical body examinations are unpleasant Psychiatric disease is a sensitive subject Psychiatric disease is hard to talk about Psychiatric disease is stigmatizing Faeces sampling is unpleasant I am feeling ashamed of my disease
	Worry of doing something wrong	Might not make it to the appointment Might not fulfil the study
	Not getting anything in return	There is nothing in it for me It will be a while before we see any results Unwilling to contribute prior to having received any help
2. Reasons to participate in research	Altruistic reasons	Helping others by contributing to science feels good Helping researchers to fulfil their study To make a difference Contribute to improving health care
	Personal reasons	Helping myself by contributing to science Positive attitude towards research Getting a reward Having a personal relationship to the research field
3. Changeable barriers to research participation	Alter the procedure of research information and request	Invitation to research should take place at a later time, when: feeling more secure at the clinic when treatment has started when feeling better Invitation to research participation should be performed by someone who knows the patient Simplify the information Ask if there are any questions The procedure should not include physical body examination, such as weight checks Do not push patients to participate
	Alter the study set‐up	Simplify participation make it possible to finish all examinations/samples straight after the invitation, so that no more visits are needed Give participants something in return: something material feedback results appreciation
4. General attitudes and thoughts towards research	Research is a good thing	Research is necessary Research is important Research contributes to better health care in society Research has helped many people Research has helped me
	Research can be manipulated	Researchers can choose to only present the findings that they want to see/show, and exclude others The selection process of participants can be biased, both consciously and by mistake Maybe the study is not randomized, and genetic data can be used to show/prove the result the scientist hopes for
	Research controls too much in health care	Medications and methods that don't have scientific support are sometimes denied or not recommended (as alternative methods) Research represents the majority – not the unique patient
5. Attitudes and thoughts towards psychiatric research	Research in psychiatry is a good thing	Willingness to contribute to psychiatric research increases, since you can relate to this field Research in psychiatry is important Positive feelings about the fact that society is trying to learn more about one's problems/disease Psychiatric research helps people
	Difficulties related to honesty among participants	It is hard to share one's problems Psychiatric illness sometimes feels taboo Psychiatric illness sometimes feels stigmatizing
	Mental illness might affect decision‐making capacity	You can be cognitively affected by psychiatric illness You might have a hard time taking in and understanding information when you are under psychiatric stress You are not in your ‘right mind’ when you have a psychiatric disease Patients with psychiatric illness might suffer from lack of insight, which can lead to accepting tasks that you do not actually have the time or energy for Patients under psychiatric stress might have a weaker mind at the time and be easy to manipulate
6. General attitudes and thoughts towards biobanks	Biobanking is a good thing	Biobanks are necessary Biobanks will lead to better health care Biobanks can help answer questions and find problems and solutions in medicine
	Biobanks do not violate privacy	Biobanking does not involve risks As long as the given information is correct – privacy is not violated Why would you care about someone saving your genetic data? There is no reason to think that donated genetic data will be handled improperly The only thing unpleasant about donating blood samples for banking is leaving the blood sample
	Biobanks violate privacy	It may be unpleasant to know that another person has access to my biological data
	Other people's attitudes towards biobanking are negative	Others probably think that access to one's genetic data might violate privacy (‘but I don't’) Many have a lack of trust in scientists/research (‘but I don't’)

#### 3.2.1 Reasons to not participate in research

In the subcategory *affected by disease,* fatigue and energy loss constituted a major obstacle to research participation. Several respondents with symptoms of depression described that everything beyond the absolute vital tasks of daily life represented a big load. Other disease‐related reasons for not participating were social anxiety and planning difficulties in neuropsychiatric disorders.

I didn't have any energy. I felt that I barely, just barely, managed the pressure from school, nothing, nothing more, no further distractions, nothing more, I just had to focus on one thing. (Patient 1, female)

The fact that *participation takes time* posed a problem for some respondents, especially if attending university studies; others reported increased stress due to multiple tasks.

In the subcategory *uncomfortable,* lack of trust, fear of disclosed information not being kept secret or used for another purpose, discomfort during sampling, for example venous puncture, and the fact that the area of ‘psychiatric diseases’ is often experienced as a sensitive subject were reported. Some respondents experienced their disease as stigmatizing and shameful. Examination of body weight and height was also considered unpleasant among patients with a negative body image. The feeling of being ‘attacked’ when the question and information about research participation were brought up was mentioned several times as an uncomfortable event. Some respondents expressed it as offensive:

… I felt a bit… yeah, well it was a little impersonal. Um… because I felt so depressed, and then someone came and asked, ‘oh, but do you want to participate in this thing, it would be great’… so … it was very… I felt terribly bad, you can't ask something like that… (Patient 3, female)

*Worry of doing something wrong,* such as not fulfilling the study requirements or performing incorrectly, was also reported as an inhibitive factor. Agreement to participate was a too big obligation for them when they knew they had difficulties to carry through things in life.

…and I didn't really trust that I would be able to submit all tests and samples. Because I had difficulties to get things done, and I didn't want that to affect the results either…and in that case I would have felt bad… (Patient 6, male)

*Not getting any reward* was an issue raised by the respondents. Participants wanted to benefit personally from the study. Some described this as getting the opportunity to other treatments but also being informed about the study results in exchange for participation.

I had to take a lot of my time, at a time when I was not well, and contribute to a study without being offered any extra treatment or help. I don't know, it is probably egoistic, but that is how I felt. (Patient 5, female)

#### 3.2.2 Reasons to participate in research

Examples of *altruistic reasons* were as follows: helping others by taking part in something that may lead to better medical care and benefit scientists in their research. Both of these factors were said to produce a positive, satisfying feeling, which would *‘enrich karma’* (quote, female). *Personal reasons* reported were curiosity, helping oneself to obtain better medical care, trust in researchers and a positive attitude towards research in general. Receiving some kind of reward was also commonly mentioned as a reason to participate. Not only material objects were mentioned, but for instance, the importance of receiving reports of the study results, appreciation and a ‘thank you’ was raised.

Yes, exactly. Maybe you can show the outcomes, like, ‘well we've done this much now, thank you for participating,’ kind of. (Patient 7, male)

#### 3.2.3 Changeable barriers to research participation

Several participants said it was inappropriate that the time‐point for research information was during their very first visit to the clinic, which, for some, had been very emotional. The majority proposed that *the procedure of research information and request* should take place later, when a more secure relationship with the health‐care professionals had been established, preferably performed by someone known.

But I think that, well, in my case… instead of asking… everyone… maybe you should ask people who have been at the clinic a little longer, I think, when they are in a place where they feel quite comfortable, and welcome. The question should come from someone you already know and who you share stuff with… (Patient 6, male)

Easily understandable information in the research invitation and encouragement to ask questions were also described as changeable factors that could promote participation. To avoid uncomfortable situations, it was recommended to obtain a sense of your patient and his/her problems/illness prior to requesting research participation.

In the subcategory *alter the study set‐up*, increasing simplicity was labelled as a changeable factor by many. Respondents reasoned that if participation had been more time efficient and comfortable, they might have joined. They also recommended excluding certain sample methods involving blood and faeces sampling.

It had been facilitating if you had taken all tests then och there, and it would have been over and not that you had to do extra things. I don't know if you had to be fasting, I don't know what tests there were… (Patient 3, female)

#### 3.2.4 General attitudes and thoughts towards research

Most respondents expressed positive attitudes towards research and spontaneously reported that ‘*research is a good thing*’. Science was also described as necessary, important and a contributing factor to better health care in society.

However, more negative thoughts also emerged, for example suspicions that *research can be manipulated* to achieve the results that scientists want to see. Doubts about selection processes were expressed, as were suspicions that researchers could choose to present only some study outcomes and ignore others if they were pointing in an undesirable direction. In the subcategory *research controls too much in health care,* it was perceived as dissatisfactory that some treatments are denied/not recommended to patients if they have not received scientific support, such as alternative medical practices. One respondent found it problematic that research does not represent everybody, only the majority, which leads to some patients being prescribed medications without an individual effect.

Well, about that… it's very easy to prescribe it, and it's very easy to think, ‘well, research shows that most people feel better with this…’ But, you know, there are a ton of people who don't. Medications are handed out to so many people who shouldn't have them, it… it's… well, a little bit like… maybe there are other things you can try as well, because it… it's a simple thing to prescribe something just because research says you should, but for some people, you shouldn't. (Patient 5, female)

#### 3.2.5 Attitudes and thoughts towards psychiatric research

The majority stipulated that *research in psychiatry is a good thing* and explained that this opinion was based on the fact that they could relate to it, and also benefit from it themselves.

Yeah, but… like I said, I'm in favor of this stuff. It makes me happy that this research is actually about something that concerns me personally. (Patient 7, male)

Pessimistic thoughts also emerged; some respondents suspected that patients with mental illness in general might have *difficulties being honest* in interviews and questionnaires because of shame, or not wanting to share about sensitive topics and problems.

…I think it is difficult to be totally honest when you report on a questionnaire. It might be that you respond imprecisely. I don't know how correct the filled in answers will be. On the other hand, for some people it might be easier, if it is difficult for them to talk about things, and easier to fill in a paper… (Patient 6, male)

The participants also discussed the fact that *mental illness might affect decision‐making capacity*. This can lead patients to accept tasks that they do not have the time or energy to accomplish. Further, anxiety disorders and depressive symptoms were described as potentially affecting cognition, especially immediately after the first visit to the clinic, when they might have opened up and exposed their problems to someone they do not know for the first time.

The disease can make you feel ‘lost in the world’ and potentially ‘weak and easily manipulated,’ since you aren't in you right mind. (Patient 9, female)

However, all respondents answered ‘no’ when asked whether they consider the procedure unethical.

#### 3.2.6 Attitudes and thoughts towards biobanks

Several positive views about *biobanking being a good thing* were mentioned; it was described as an exciting field that leads to better medical care. Interestingly, the majority suspected that *other people's attitude towards biobanking is negative,* but stated that they personally approved.

If you have problems trusting people then maybe you don't trust that they will…, that they who have asked you to participate will do exactly as…they had said. But, I don't think so… (Patient 6, male)

Regarding privacy, opinions differed. Most respondents did not see it as a problem at all to contribute biological samples for research purposes (*biobanks do not violate privacy)*, while some described it as uncomfortable. Having genetic material banked somewhere for many years with no actual control over how and when it would be used would *violate privacy* for some respondents.

No, not really (violating integrity). All the same, I have been…to doctors and all such, during a longer period of time so… such things are not really very important for me… (Patient 4, male)

Yes, but it is that (leaving biological samples) felt uncomfortable, sort of. …since I was younger I have had a picture in my head that…it is not good for me, sort of, someone would be storing…yes, but, kind of …, yes, but in biobanks or something, something from me. (Patient 7, male)

## 4 DISCUSSION

This study describes the rationale behind a patient's decision to not participate in psychiatric research and identifies several factors that may have influenced that decision. The quantitative study found the most common reason for not participating in research was being ‘too tired/too sick to participate’. Fear of needles and lack of time were also common reasons. Interestingly, lack of trust was less often reported as a barrier to research participation. The result from the qualitative study was more nuanced, but confirmed the findings above. Despite declining to participate, patients’ attitudes towards research, especially in the psychiatric field, were mainly positive. Many suggested that it would have been easier to join UPP if participation included some kind of personal reward and if it had occurred at a later time‐point when symptoms were fewer and a personal relationship had been established.

Lack of energy was the main reason for declining to participate and is a common symptom in psychiatric disorders. Our results indicated that this symptom might explain a large part of the generally low participation in psychiatric research. General anxiety and depressive symptoms are correlated with a lower tendency to agree to research participation among patients with somatic disease as well.^39^ When understanding the sample bias introduced by this factor, it is important to emphasize that it is *psychiatric symptoms* – rather than psychiatric disorders – that seem to constitute a barrier to research participation.

The ability of patients with a psychiatric illness to understand information and make decisions has frequently been discussed.^27^, ^30^, ^31^ This study suggests that patients with a psychiatric illness consider these abilities to be affected *themselves*. There was however no indication, even when asked directly, that these patients considered inquiries about research participation unethical. This provides valuable information; in recent years, research ethics have been frequently discussed, but patients’ own opinions are rarely available.^29^

The invitation to participate in research was experienced negatively. First, the timing of the request in conjunction with the patient's first visit was criticized, and second, being asked by an unknown person was criticized. Although previous studies have given little attention to this aspect, it has been mentioned that not knowing the scientist/recruiting clinician has a negative effect on willingness to participate.^37^ Furthermore, trust in clinicians and particularly the patient's treating doctor plays an important role in the research recruitment process.^10^ In UPP, the patients are asked by a research nurse and not by the clinician.

In the quantitative study, several respondents reported that they did not feel a lack of trust themselves – but thought that others probably did; for example, they raised a fear of their personal data and submitted biological samples being handled differently than what had been promised. These concerns have also been reported in previous studies.^22^, ^40^ Furthermore, suspicions were raised that research results can be manipulated to show what the research group wants to see. Differences in outcomes between questionnaires and interviews may have more than one explanation. In a questionnaire, pre‐formulated statements cannot capture every possible thought and reflection among respondents. Another factor to consider is that the number of participants differed in questionnaires vs interviews. Furthermore, the questionnaires pertained to participation in UPP, whereas the interviews also discussed research participation *in general*.

The majority of the survey respondents were women, while the number of female and male respondents was equally distributed in the interviews. In research involving patients with mental disorders, however, women participate more often.^41^, ^42^ Gender bias is not reflected in participation in biobank studies in general.^43^, ^44^ However, the probability of participation in *biobank research among patients with mental disorders* is larger among women (in certain categories).^45^ It may be speculated that men with psychiatric disorders lack trust in research to a greater extent and therefore choose to abstain more often, but this was not evident in our results and requires further research.

Another pattern that can be discerned from the interviews is that research participants would be encouraged by something in return for their participation, including not only material things, but also appreciation and confirmation. For example, letters with information about how the study is proceeding, if progress has been made, and a ‘thank you’ for taking part were proposed ideas. Participants in a previous study^24^ also wished to be informed of the study results. This type of reward may better indicate the value of their participation. This wish must be considered in relation to routines for data protection. If all data concerning participants' details is destroyed after collection, as it is in many studies, it will not be possible to contact the participants.

The purpose of this study was to investigate factors that prevented young patients with a psychiatric illness from participating in research. Although the study included a quantitative portion, literature on the field is inconclusive; therefore, the study aim was framed as descriptive rather than hypothesis‐testing. Recruitment was rendered difficult by the fact that the target group (patients who rejected UPP) had already declined to participate in research. No registers of ‘UPP decliners’ existed, complicating the delivery of questionnaires. Instead, the staff was instructed to ask *all* visitors whether they had accepted or rejected UPP. The patient who reported rejection could then choose to answer the questions or not, and no oversight of their participation status was conducted. The reasons for declining participation in both UPP and this study thus remain uninvestigated. The study has several other limitations. Ten respondents choose to take part, and all were included in the interview study. Data saturation could therefore not be used in the recruitment procedure. This is a limitation and may lower generalizability of the findings, as a larger sample of respondents might have led to additional information. From an ethical view, the number was seen as acceptable, since asking patients that previously declined to participate in UPP about taking part in this interview study was delicate. Even if we don't know the number of patients asked to participate in the questionnaire study, we know that there are over 500 UPP decliners, and therefore, participation rate is this study is around 10%. We don't know how representative they are of all decliners. Furthermore, the deductive approach in the analysis of the interview data may have led to interpretations restricted to the predefined categories and missing respondents’ voices. To minimize this, the whole text was used when searching for units of meaning bearing the aim of the study in mind. The questionnaire also has some limitations; first, the Likert scale is positively skewed, as the midpoint is not neutral. This may have affected the data quality as participants could have assumed that the midpoint was neutral, and as the response options are not at equal intervals, it might be unclear what level of agreement/disagreement points 2 and 4 reflect. However, there were textual descriptions to guide the patient. An especially important strength of this study is the focus on young adult very sick psychiatric patients. It is crucial to include in this group psychiatric research, and knowledge about potentially removable barriers for participation is paramount. It is clear from this study that the major barrier for many patients who declined participation in UPP was the strong influence of symptoms due to psychiatric illness. This barrier is at odds with the need to include patients at baseline, where symptoms are often most acute, in order to follow state effects of disease and treatment response. Encouragingly, attitudes towards and trust in psychiatric research were generally positive. Most patients approved of being asked about participation, which is an important finding as well as their suggestions for improvements, such as simplification of procedures and information, the timing of the request and greater personal rewards for patients.

## CONFLICT OF INTEREST

None of the authors have any conflict of interest.

## Data Availability

Data sharing is not applicable to this article as no new data were created or analysed in this study.
